# Listening Deeply to Indigenous People: A Collaborative Perspective and Reflection Between a Mapuche Machi and Ecologists

**DOI:** 10.1002/ece3.71914

**Published:** 2025-08-07

**Authors:** Andrea Monica D. Ortiz, Patricia Huinca Blanco, Carlos Alberto Arnillas, Anni Arponen, Marc W. Cadotte, Javiera Beatriz Chinga, Mariana C. Chiuffo, Sharon Collinge, Kadambari Devarajan, Ken Ehrlich, Marilyn Grell‐Brisk, Claudio Guevara, Rebecca W. Kariuki, Heather M. Kharouba, Tara G. Martin, Ana Carolina Prado‐Valladares, Helen M. Regan, Nicolás Santos Domínguez, Bruno Eleres Soares, Gisela C. Stotz, Ivette Ulloa Caniú, Kristiina Visakorpi, Marten Winter, Florencia A. Yannelli

**Affiliations:** ^1^ Departamento de Geografía, Facultad de Arquitectura, Urbanismo y Geografía Universidad de Concepción Concepción Chile; ^2^ Instituto de Ecología y Biodiversidad Concepción Chile; ^3^ Mapuche Indigenous Peoples Wallmapu Chile; ^4^ Department of Physical and Environmental Sciences University of Toronto — Scarborough Toronto Ontario Canada; ^5^ Department of Biology University of Turku Turku Finland; ^6^ Department of Biological Sciences University of Toronto — Scarborough Toronto Ontario Canada; ^7^ Escuela de Ingeniería en Medio Ambiente y Sustentabilidad Universidad Mayor Santiago Chile; ^8^ Grupo de Ecología de Invasiones, INIBIOMA Universidad Nacional del Comahue, CONICET San Carlos de Bariloche Argentina; ^9^ Arizona Institute for Resilience Tucson Arizona USA; ^10^ Department of Natural Resources Science University of Rhode Island Kingston Rhode Island USA; ^11^ Roski School of Art and Design, University of Southern California Los Angeles California USA; ^12^ Pitzer College Claremont California USA; ^13^ Forest Engineering, Resources and Management Oregon State University Corvallis Oregon USA; ^14^ School of Sustainability, College of Global Futures Arizona State University Tempe Arizona USA; ^15^ Department of Biology University of Ottawa Ottawa Ontario Canada; ^16^ Department of Forest and Conservation Sciences University of British Columbia Vancouver British Columbia Canada; ^17^ Fundação Instituto de Pesca do Estado do Rio de Janeiro Rio de Janeiro Brazil; ^18^ Department of Evolution, Ecology, and Organismal Biology University of California Riverside Riverside California USA; ^19^ Institute of Environmental Change & Society, University of Regina Regina Saskatchewan Canada; ^20^ Instituto One Health Facultad de Ciencias de la Vida, Universidad Andrés Bello Santiago Chile; ^21^ Center of Applied Ecology and Sustainability (CAPES) Santiago Chile; ^22^ Department of Biology Norwegian University of Science and Technology Trondheim Norway; ^23^ German Centre for Integrative Biodiversity Research (iDiv), Halle‐Jena‐Leipzig Leipzig Germany; ^24^ Argentine Institute for Dryland Research (IADIZA) CONICET and Universidad Nacional de Cuyo Mendoza Argentina

**Keywords:** biodiversity, indigenous peoples and local communities, Mapuche, traditional ecological knowledge, two‐eyed seeing

## Abstract

Indigenous Peoples are key knowledge holders and essential partners to confront global environmental crises, especially biodiversity loss. Many calls have been made to better integrate Indigenous Traditional Ecological Knowledge and Western ecological sciences. However, partnerships between these communities are complex due to power imbalances, distrust, different objectives, and injustices towards Indigenous Peoples. This raises the question of what meaningful engagement is, and for whom. These issues were discussed at a scientific workshop in Conguillío National Park, Chile. This initial encounter between ecologists and Mapuche elders, including a Machi (a Mapuche spiritual authority), has led to ongoing dialog and engagement. Responding to calls to listen deeply towards engagement with Indigenous Peoples in Western ecological sciences, we—the Machi and scientists—present our joint perspectives and reflections upon the process, drawing from Indigenous Knowledge and Western ecological sciences. Interweaving both lived experiences and scientific evidence, we document the environmental issues confronting the local Mapuche community caused by industrial developments in the territory. Our joint account highlights conflicts caused by non‐native tree plantations and the plans to construct a hydroelectric plant in the Truful–Truful watershed, which was opposed strongly by the local communities. Together with the industrial forestry plantations that cause land‐use change, the construction of this hydroelectric plant endangers biodiversity, including species of conservation significance, medicinal plants, and ultimately, the Mapuche way of life. Reflecting upon our collaboration and the process facilitated by Two‐Eyed Seeing, we illustrate that Indigenous voices and scientific evidence, together, can deepen our understanding of social‐ecological change in the territory and reveal opportunities for building trust and relationships. We highlight the importance of time, preparation for engagement, and advocating for change in knowledge partnerships in the ecological sciences. Learning from our collaboration, we call upon our communities to continue listening, engaging, and advocating for Indigenous representation in ecology.

## Graphical abstract

Two‐eyed seeing offers a way to see with both Indigenous Traditional Ecological Knowledge and Western ecological sciences. In this collaborative perspective piece between ecologists and a Mapuche spiritual authority (a Machi), we interweave lived experiences and ecological evidence to present the alarming social‐ecological devastation and conflict in Mapuche territory in Chile. We offer learnings from our collaboration and call upon our communities to respond to the call for deep listening and meaningful engagement.

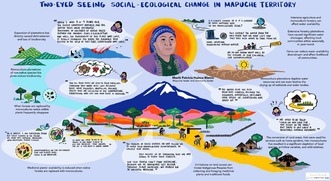

## 1 Introduction

The vital contributions of Indigenous Peoples in the protection, conservation, restoration, and sustainable use of nature are increasingly recognized in science and policy, including the Intergovernmental Panel on Biodiversity and Ecosystem Services (IPBES) and the United Nations Convention on Biological Diversity (UN CBD) (Tengö et al. 2017). This includes the decision to establish a permanent Indigenous Peoples subsidiary body under the UN CBD.^1^ Such recognition is well justified, as Indigenous Knowledge and Traditional Ecological Knowledge are critical for preserving the biological and cultural diversity of our planet (Fernández‐Llamazares et al. 2021).

Many terms describe Indigenous Peoples' creative expressions (Duarte et al. 2020). These include, among many others, Indigenous Knowledge (IK), Traditional Ecological Knowledge (TEK), Indigenous and Local Knowledge (ILK) – the latter term being used in the IPBES reports. The term TEK has come to refer to Indigenous Peoples' legitimate systems of knowledge production, which have empirically tested and testable understandings of the relationships among living things and their environments, though there may be differences with Western scientific approaches characteristic of disciplines like ecology (Whyte 2013). Indigenous Knowledge also includes TEK when relating to ecology (Jessen et al. 2022). Thus, throughout the text we use the term “Indigenous TEK” to emphasize Indigenous Knowledge in the ecological context.

Indigenous TEK represents generations of experiences, observations, and information on the sustainable use of planetary resources collected by Indigenous Peoples. Much of Indigenous TEK is based upon the deep social, cultural, and spiritual ties of Indigenous Peoples to their territories (Garnett et al. 2018). Many Indigenous worldviews do not see humans as separate from nature and value kinship and reciprocal relationships with other‐than‐humans, ancestors, and spirits (Lenzi et al. 2023; Raymond et al. 2023). Indigenous TEK is used for land management to guide hunting, fishing, farming, and foraging, which are activities closely linked to Indigenous Peoples' livelihoods, health, and well‐being (Ford et al. 2020; Gordon (Iñupiaq) et al. 2023).

Indigenous TEK helps protect and manage ecosystems towards better biodiversity outcomes. About 7.8 million km^2^ (20.7%) of Indigenous Peoples' lands are within protected areas, encompassing at least 40% of the global protected area (Garnett et al. 2018). There are higher levels of biodiversity and forest integrity in Indigenous‐managed lands (O'Bryan et al. 2021; Sze et al. 2022). Indigenous Peoples' traditional practices in their lands have reduced deforestation in the Amazon, maintaining forest structure and functionally richer ecosystems (Armstrong et al. 2021; den Braber et al. 2024; Fletcher et al. 2021). Indigenous Peoples across British Columbia and the Pacific Northwest (i.e., First Nations communities) have systems of salmon management that integrate stewardship, cultural, and spiritual beliefs (Atlas et al. 2021; Reid et al. 2022). Indigenous Marine Areas and action networks in Chile contribute to ocean sustainability, safeguarding local fishing communities and protecting against threats (Araos et al. 2020, 2023). These are a few examples of where Indigenous TEK and practices of Indigenous Peoples have sustained ecosystem services, while also protecting ecosystems from threats, including biological invasions (see Seebens et al. 2024).

Yet, despite the importance of Indigenous TEK, many challenges remain. Biodiversity loss due to land‐use change continues at an alarming rate, causing significant harm to both people and nature (IPBES 2019). The unsustainable exploitation of nature disproportionately affects Indigenous Peoples' health, food security, and well‐being (Redvers et al. 2023). Despite international frameworks like the 2007 United Nations Declaration on the Rights of Indigenous Peoples (UN‐DRIP) affirming Indigenous Peoples' rights to self‐determination, land, resources, and participation, their voices remain marginalized in environmental decision‐making processes (Robinson et al. 2021). Indigenous Peoples continue to face significant threats from colonialism, globalization, land theft, short‐term economic interests, biopiracy, and violence (Cottrell 2022; Fernández‐Llamazares et al. 2021). These challenges have left enduring legacies of exclusion and poverty. For instance, land dispossession created the groundwork for the current conditions in which Indigenous Peoples face greater vulnerabilities and increased exposure to climate change in the United States (Farrell et al. 2021).

Many scholars and practitioners have called on ecologists and environmental scientists to engage with Indigenous Peoples and Indigenous TEK as part of efforts to address the biodiversity crisis (Gann et al. 2019; Ogar et al. 2020; Reyes‐García et al. 2019; Robinson et al. 2021). However, they also caution that this engagement cannot be reduced to the “integration” of Indigenous TEK into Western scientific paradigms; it requires attention to questions of epistemology, that is, the study of the nature of knowledge itself, how it is formed, and its limitations. Western sciences, including ecology and evolution, have long drawn insights from Indigenous TEK to understand, among others, population trends, ecosystem functions, and biogeographic patterns (Jessen et al. 2022). However, the settler‐colonial institutions of Western ecological sciences are complicit in propagating the belief that only Western sciences are valid and/or are superior to Indigenous and other ways of knowing (Gazing Wolf et al. 2024). The engagement of ecologists and environmental scientists with Indigenous Peoples includes historical injustices, harm, and extractive activities carried out towards Indigenous Peoples, their bodies, and their territories in the name of science (Jessen et al. 2022).

Efforts to “integrate” Indigenous TEK with Western science thus risk the appropriation of Indigenous Knowledges and losses in cultural identity solely to benefit the researchers or to meet formal project requirements (Chapman and Schott 2020). All of these (mis)practices lead to distrust of Western scientific approaches by Indigenous Peoples' communities, despite how well‐intentioned these efforts may be (see Bozhkov et al. 2020; Kater 2022; Lauter 2023; Morales et al. 2021), creating a greater imperative for building true partnerships based in trust. A more equitable and respectful approach requires dismantling colonial biases in science, centering Indigenous leadership, and fostering collaborations that genuinely value Indigenous TEK, voices, and sovereignty.

Recognizing these challenges and barriers, this contribution seeks to document the experiences of deep listening and trust‐building between ecologists and elders of the Mapuche Indigenous Peoples community in South‐Central Chile. Although we use the term‘ecologists’, the Western co‐authors are people from diverse backgrounds, including other natural, physical, and social sciences and the arts. This collaboration began during a workshop held in Conguillío National Park, in *Wallmapu*,^2^ the territory of the Mapuche Peoples, where ecologists and Mapuche elders from Melipeuco and Cholchol in the Araucanía region came together to share knowledge and experiences. The initial exchange between ecologists and the Mapuche elders at the workshop revealed a shared commitment between non‐Indigenous ecologists and the Mapuche to caring for nature. The ideas in this piece grew out of the relationships and reflections that followed.

Ongoing dialog between a Mapuche Machi^3^ [PHB] from the Juan Colipi Huenchunao community and one of the environmental scientists [AMDO] helped shape the direction of this work. Inspired by the Mi'kmaq principle of Two‐Eyed Seeing or *etuaptmumk*, which values the strength of multiple perspectives, this piece serves as both a collaborative reflection and a call to action. Together, we seek to make an urgent message visible: the land is in crisis, and the social‐ecological destruction taking place in the territory remains largely unknown to the Western world. Honoring the importance of invitation and reciprocity within Mapuche traditions (see Guzman and Krell 2024), this article brings together TEK and Western science to highlight social‐ecological challenges in the Truful–Truful watershed and the need for new kinds of listening, partnerships, and responses to the environmental crises. Specifically, this collaboration aims to: (i) Respond to the invitation to listen deeply and join the perspectives of TEK and Western scientific tools to characterize, from both ways of knowing, the environmental issues faced by the communities in this territory, combining both Indigenous TEK and scientific evidence as a way of listening, engaging, and advocating for change; (ii) represent a commitment to continue to respond to the call to listen deeply to and engage with Indigenous Peoples in the ecology and environmental sciences, ensuring their voices are heard and elevated through their own perspectives and lived experiences; and (iii) explore Two‐Eyed Seeing as a means to open respectful dialog and build trust between different ways of knowing in the context of social‐ecological change.

## 2 Methodology

Two‐Eyed Seeing emphasizes the integration of Indigenous and Western ways of knowing. Introduced by Mi'kmaq Elders Albert and Murdena Marshall and Dr. Cheryl Bartlett, this principle advocates for learning to see from one eye with the strengths of Indigenous TEK, and from the other eye with the strengths of mainstream scientific knowledge, using both together for the benefit of all (Bartlett et al. 2012). Two‐Eyed Seeing and the transformation pathways advocated by Indigenous scholars in the ecological sciences (Gazing Wolf et al. 2024) inspired us to speak together and make visible the social‐ecological challenges that the territory faces.

This approach was adopted during the exchanges between 27 ecologists from 15 countries and four elders of nearby Mapuche communities during the workshop held in February 2024 in Conguillío National Park, Chile. The workshop was designed to reflect on the collective values and responsibilities of ecologists in the face of ecological crises, and how this can be transformed from research into action (Yannelli et al. 2025). The organizers sought to ground the gathering in the territory by inviting elders from nearby Mapuche communities, relying upon local contacts in Melipeuco (Chile) for this. Welcoming the ecology workshop participants into their territory, the Mapuche elders shared their experiences of environmental changes in this area affecting their water, land, and lifeways. The Mapuche elders shared how the land‐use changes have caused the conversion of native forests to industrial tree plantations, which in turn caused significant harm to their people because of the loss of access to their land and the transformation of landscapes. At the gathering, conversations between ecologists and Mapuche elders centered on the controversy and concern over a planned project to install a hydroelectric power plant in the Truful–Truful watershed, to which Conguillío National Park belongs. The ecologist co‐authors were urged to bring this struggle to the international scientific community to draw attention to the case and lend support towards its rejection, specifically to find scientific work to support the opposition of the community to the project. Following this invitation and request, the co‐authors worked together to document the lived experiences and narratives of social‐ecological change.

While the initial focus of the workshop was on academic dialogs within the ecological community, the gathering organically evolved into an opportunity for deeper engagement, upon invitation by the Machi co‐author [PHB] to the co‐authors based in Chile. Recognizing our own power dynamics, different backgrounds, and roles that we brought to the conversations, the process of developing this perspective was guided by close collaboration and communication between the lead and Mapuche co‐authors, shaped by an ethic of mutual respect and meaningful engagement. Through these smaller, more reciprocal conversations, the ecologist team also reviewed studies from Western ecological literature based on the Machi co‐author's first‐hand insights to produce scientific evidence matching lived experiences, as requested by the community. This perspective is thus a product of respectful listening and scientific collaboration between all the authors. To honor and respect their role and insight, the Mapuche co‐author PHB is referred to in the third person. This is also to preserve narrative clarity and distinguish their lived experiences in the territory. The next section provides more context and history about the territory and its social‐ecological conflicts.

## 3 Results and Discussion

### 3.1 The History of the Mapuche in the ‘Forestry Territory’ of Chile

Historically, as well as at present, many communities of the Indigenous Mapuche Peoples reside in *Wallmapu*, an expansive territory that spans the countries of Chile and Argentina. The Mapuche, whose name means “people of the land” in Mapudungun,^4^ are the largest Indigenous group in Chile. The Mapuche Peoples include the *Pehuenche* and *Lafkenche*, among others.^5^ The Mapuche have a long history of resisting colonization, first by the Inca Empire, then by European invaders, and later by the Chilean State (Alberti et al. 2023; Meza 2009). The violent land displacement during the “pacification” of the Araucanía region—the heartland of the territory—reduced Mapuche land from ten million hectares to 500,000 ha (Warren 2017). The Mapuche are deeply affected by colonization and persistent land disputes, resulting in deep‐rooted conflicts with the Chilean State. There are many active conflicts that affect Indigenous Peoples in Chile, related to mining, thermal and hydropower projects, fishing and forestry projects (Delamaza et al. 2017).

In South‐Central Chile, the most visible conflict is with the industrial forestry model, which has significantly modified landscapes, creating a forestry territory in Mapuche land (Carrasco Henríquez and Mendoza Leal 2021; Ortiz et al. 2024). The industry expanded rapidly in the 1970s during Augusto Pinochet's dictatorship, which reversed land reforms and changed traditional rural practices (Robles 2020; Torres et al. 2015; Hofflinger et al. 2021). Vast areas of native forest were transformed into fast‐growing plantations of non‐native species, predominantly Monterey pine ( *Pinus radiata* ) and eucalypts (*Eucalyptus* spp.). It has been argued that conservation and forestry science served as tools for extending state governance into a frontier territory through the promotion of plantations (Klubock 2014). Industrial tree plantations in Chile negatively impact native biodiversity and ecosystem services, increase wildfire risk, change hydrological basin dynamics, and, as these species are invasive, they invade native ecosystems, further degrading the landscapes (Alvarez‐Garreton et al. 2019; Braun et al. 2021; García et al. 2018; Heilmayr et al. 2016; Langdon et al. 2023; Simberloff et al. 2010; Úbeda and Sarricolea 2016). Chile's forestry model is also a key driver of the displacement, increased poverty, and negative impacts on the health and well‐being of the Mapuche and local communities (Andersson et al. 2016; Beltrán‐Véliz et al. 2023; Braun 2021; Garrido and Alarcón 2023; Meza 2009; Schmalz et al. 2022; Torres‐Salinas et al. 2016).

In addition, Chile has a unique system of private water rights that prioritizes economic interests and has led to significant overexploitation and negative ecological impacts on rivers and watersheds (Bauer 2015; Budds 2004, 2020; Larrain 2012), including for hydroelectric energy.

### 3.2 Dams and Hydroelectric Plants as Sources of Conflict: Ralco and Truful–Truful

Hydroelectric developments are a major driver of social and environmental conflict in Chile, especially for Indigenous Peoples. Hydroelectric projects disrupt hydrological cycles, harm relationships between people and rivers as ceremonial spaces, and endanger water sources and medicinal plants essential to Mapuche cultural and spiritual life (Kelly 2019). Indeed, conflicts over the exploitation of hydrographic basins to produce energy outrank the number of reported forestry conflicts in a review of socio‐territorial conflicts (Delamaza et al. 2017). A historical example of this is the Ralco dam, which was built in 2004 despite significant Mapuche and environmental mobilization (Hohl 2018; Lorenzo 2002). The Ralco dam construction in the Biobío River is seen as a turning point in the history of the hydroelectric sector in Chile. It caused significant controversy, resistance, and violence between Mapuche communities and the Chilean state, especially for the Pehuenche Peoples of the territory where it was built. The Chilean state supported the foreign investment by the Spanish company ENDESA, in the name of energy and development. The movements that formed to resist Ralco questioned whether the negative social and environmental impacts caused by this type of development were justifiable (Hohl 2018). Apart from the displacement of families, the construction of the Ralco flooded 3936 hectares in Pehuenche territory, including many sites of cultural significance, such as community cemeteries, places of high biodiversity and scenic value, and those fundamental to Mapuche life (Orellana 2005). These losses had lasting consequences in the territory and the relationship between the Mapuche and the state.

**FIGURE 1:**
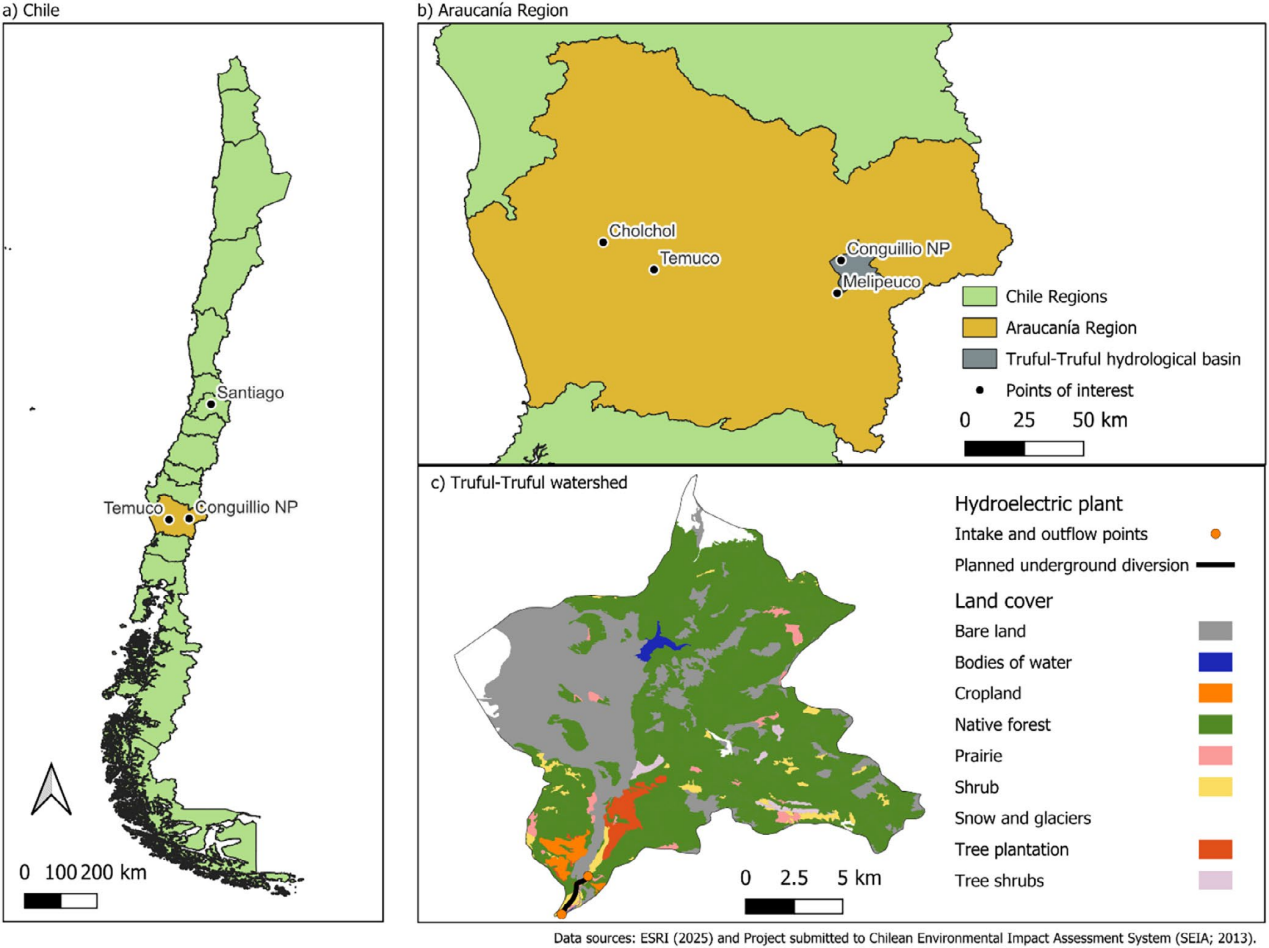
A map of Chile showing (a) continental Chile; (b) the Araucanía region, to which Conguillío National Park belongs, as well as the municipality of Melipeuco where the authors met. Temuco is the regional capital, and Cholchol is the municipality of the Machi co‐author. (c) Shows land cover of the Truful–Truful watershed, which is threatened by the plans for a run‐of‐the‐river hydroelectric plant.

The Truful–Truful watershed, nestled in the Andean foothills near Melipeuco (Figure 1), is the site of a similar and recent conflict over a proposed run‐of‐the‐river hydroelectric project. The proposed project, “El Rincón” run‐of‐the‐river hydroelectric plant (*Central Hidroeléctrica de Pasada El Rincón*) was submitted to Chile's Environmental Assessment Service in 2013. It aimed to generate 11 Megawatts of energy by diverting a portion of the river's flow through a 3‐km underground conduit. There were strong criticisms of the ecological assessment of the project, which planned the underground installation of intake structures that would have increased the likelihood of contamination and degradation of the Truful–Truful River (Salinas 2023; Personal communication with F. Salinas, 7 May 2024). The river provides essential ecosystem services to the Conguillío National Park, which is part of the Araucarias UNESCO Biosphere Reserve (UNEP‐WCMC and IUCN 2025). Although the general perception of run‐of‐the‐river hydroelectric plants is that they have comparably little or no impacts compared to larger dams, they can alter the natural flow of rivers and negatively affect the ecosystems upstream and downstream, changing vegetation, fish composition, and water quality, among many other impacts (Kuriqi et al. 2021).

**FIGURE 2:**
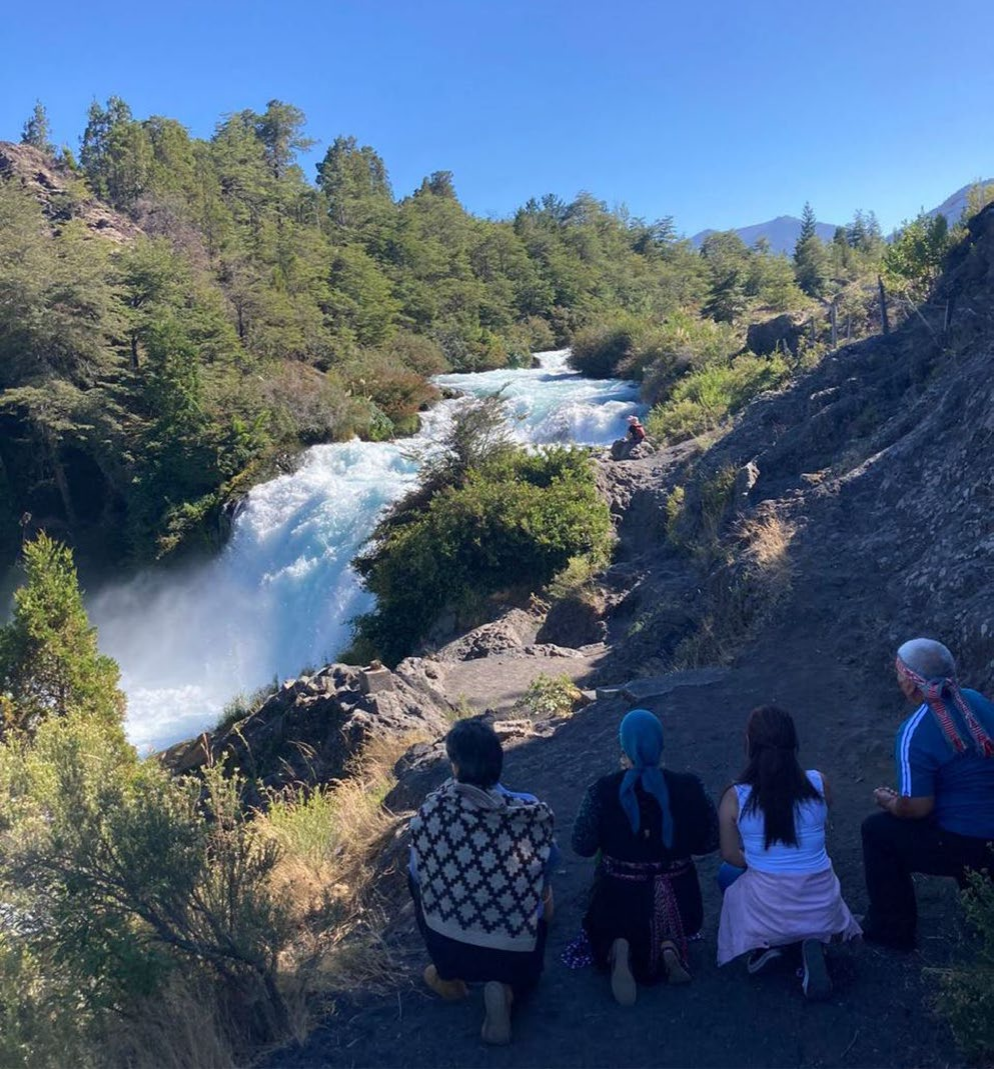
Mapuche elders asking for permission and blessings from the spirits for our meeting in their territory. Truful–Truful Gorge, Melipeuco, Wallmapu (South‐Central Chile). Photo by C. Prado‐Valladares.

Since the submission of its environmental impact evaluation in 2013, the project was met with sustained opposition from Indigenous organizations, local residents and leaders, and environmental advocates. Significant concerns included the impacts on the river's ecosystem, its cultural and spiritual significance, and local biodiversity (Huneeus et al. 2021). The Truful–Truful River and its surrounding landscape are of cultural and spiritual importance for the Mapuche. As the Machi co‐author of this paper shares, the Truful–Truful is a place of healing and spiritual practice (Figure 2). Initially, the Chilean Environmental Assessment Service rejected the project in 2018 (Huneeus et al. 2021). However, the rejection was overturned when the project proponent appealed; their appeal was accepted and the project approved by a ministerial‐level committee in 2021. The El Rincón case was brought to the Chilean environmental court, and after years of deliberation the project was finally rejected in March 2025 (Tercer Tribunal Ambiental de Chile 2025).

This decision can be considered favorable. However, it underscores how even modern systems of environmental impact assessment that include citizen participation and mechanisms for consultations with Indigenous Peoples can still approve projects that can have significant ecological and social impacts on Indigenous Peoples—revealing persistent deficiencies in the system. The ultimate rejection of the project came from the environmental court, bolstered by the sustained efforts by Mapuche and local communities. The case of Truful–Truful underscores the importance of collaboration between local communities, environmental organizations, and the local municipality to oppose conflictive projects (Huneeus et al. 2021), but also the ecological and scientific evidence that raises awareness on the case (e.g., Salinas 2023). The spiritual perspective from the Machi co‐author also offers insight into the lived experiences and long‐term impacts of environmental change. Her testimony highlights how industrial developments such as forestry plantations have already disrupted local biodiversity and traditional practices, and how projects like the El Rincón hydroelectric plant threaten to further erode the cultural and ecological integrity of the territory. These reflections offer an understanding of how these social‐ecological changes are experienced, remembered, and resisted within the territory.

### 3.3 Lived Experiences of the Machi: Social‐Ecological Changes in the Territory

**FIGURE 3:**
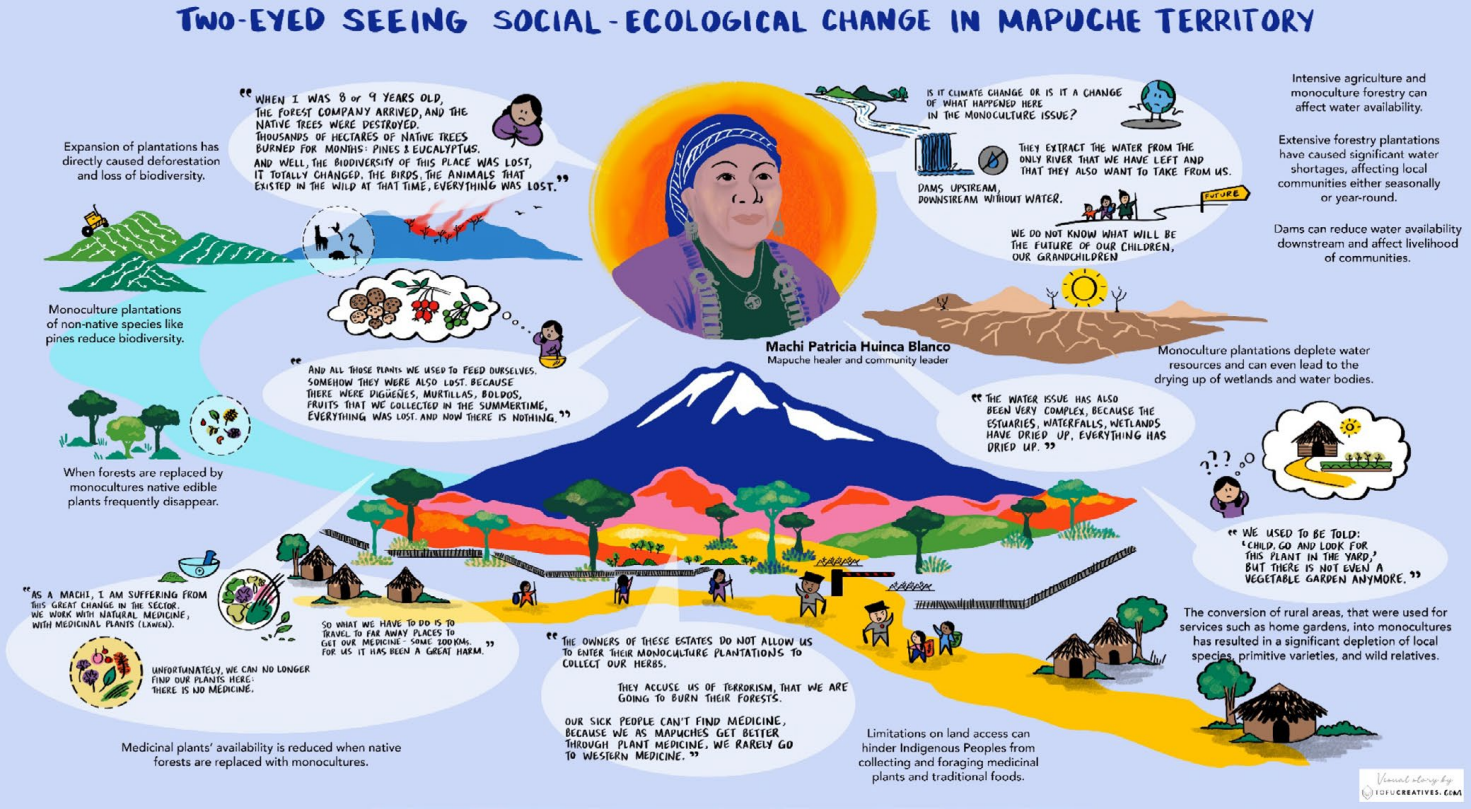
Social‐ecological change from the perspective of a Mapuche Machi (healer and community spiritual leader) in the industrial forestry landscapes of South‐Central Chile, weaved together with scientific studies in Table 1 to see social‐ecological change through Indigenous and Western scientific perspectives.

The Truful–Truful is considered a sacred river by the Mapuche, home to many *ngen* (spirits) and *newen* (forces).^6^ Many Machis come to the river to find and gather medicinal plants (*lawen*) and for spiritual cleansing. In the Mapuche worldview, spirituality is deeply relational, extending to family, community, and territory. This is expressed through *küme mogen* (good living), a principle of balance and mutual respect between people and nature (Meza‐Calfunao et al. 2018). While many understand *lawen* as herbal remedies, health is better understood as harmony between people and nature. In our exchanges, the Machi co‐author [PHB] shared memories and grief over how the territory has changed and how this affects her practice as a Machi (Figure 3). She recounts that, as a child, the arrival of a forestry company led to the burning of thousands of hectares of native forest and its replacement by monocultures. This devastation resulted in the loss of local biodiversity, including birds, wild animals, and traditional foraged foods like mushrooms and berries.

These changes have disrupted her practices as a spiritual and medicinal healer, as she is forced to travel longer distances to access *lawen* that she would have normally been able to find in her own backyard. The Machi also shared that now, key water sources, such as estuaries, waterfalls, and wetlands, have dried up. She wonders whether this is truly due to “climate change”, Although she was unable to be listed as a co‐author, she shares a personal commitment to the collaboration. or rather, the impacts of extractive industries in the territory that have used all the water and threaten to harm the river more with the hydroelectric plant. The homes of her family have also been affected by wildfires that started in the eucalyptus plantations near their community, affecting the grazing land for their small flock of sheep. The Machi's account and perspective highlight the deep cultural and ecological impacts, pain, and harm caused by these changes in the territory, as well as the uncertainty of the future. Table 1 brings together Indigenous perspectives on territorial changes alongside scientific studies documenting social‐ecological transformations, developed in accordance with the elders' request from the initial workshop meeting, our conversations together, and guided by the Two‐Eyed Seeing approach.

**TABLE 1:** Indigenous Peoples' perspectives of changes in the territory and scientific studies that also document the significant social and ecological changes in the South‐Central region of Chile.

Local perspective	Scientific references
Loss of biodiversity in the territory due to monoculture plantations	The expansion of plantations in Chile has directly caused deforestation and the loss of biodiversity (Nahuelhual et al. 2012) Monoculture plantations of non‐native species, like pines, reduce native biodiversity and support the spread of invasive trees beyond plantations (Corley et al. 2018; Franzese et al. 2017; Lantschner et al. 2011; Wang et al. 2022)
2 Loss of native edible plants	When forests are replaced by monocultures, native edible plants frequently disappear (Barreau et al. 2016, 2019; Monterrubio‐Solís et al. 2023)
3 Changes in the accessibility of medicinal plants (*lawen*)	Medicinal plant availability is reduced when access is limited to native forests, which have been replaced with monocultures (Barreau et al. 2016; Beltrán‐Véliz et al. 2023)
4 Loss of community and home gardens	The conversion of rural areas, that were used for services such as home gardens, into monocultures has resulted in a significant depletion of local species, ancient plant varieties, and wild relatives (Galluzzi et al. 2010; Sunwar et al. 2006)
5 Water scarcity in forest plantation watersheds	Monoculture plantations deplete water resources (Alvarez‐Garreton et al. 2019; Balocchi et al. 2023; Huber et al. 2008; Lara et al. 2009) and can even lead to the drying up of wetlands and water bodies (Mansilla‐Quiñones et al. 2024) leading to the loss of important ecosystem services (water, habitat for species)
6 Effects of land‐use change, including dams, on water availability	Intensive agriculture and monoculture forestry can affect water availability (Balocchi et al. 2023; Lara et al. 2009; Scanlon et al. 2007) Extensive forestry plantations have caused significant water shortages, affecting local communities (González‐Hidalgo and López‐Dietz 2020) Dams can reduce water availability downstream and affect livelihood of communities (Aigo et al. 2022)

The process of listening revealed a convergence and a fuller picture of the impacts of change in the territory: both Indigenous experiences and ecological research point to significant changes in biodiversity, water availability, and ecosystems. Indeed, when the forestry companies purchased large estates in South‐Central Chile, agriculture and livestock raising were also replaced by non‐native monocultures (Carte et al. 2021). The changes to the landscape have also caused significant losses of Indigenous TEK and traditional practices, which are closely intertwined with the spiritual dimensions of the Mapuche cosmovision. Such changes are exemplified by the decline in traditional foods, including nuts from the native conifer and cultural keystone species *Araucaria araucana* (*pewen*), edible plants and *lawen* that are central to Mapuche spirituality, identity, and ceremonies. The lack of access to forests has also altered the intergenerational transmission of Indigenous TEK about these practices (Barreau et al. 2016, 2019; Fernández 2020; Monterrubio‐Solís et al. 2023).

Additionally, the expansion of forestry, along with increasing foraging and harvesting of medicinal plants, has reduced the population density of many native and endemic species. This is exacerbated by the lack of cultivation efforts and absence of regulations on foraging in Chile (Susana et al. 2011). The loss extends beyond the physical unavailability: the therapeutic efficacy of Mapuche medicine is not only based on “active agents” but is also related to their symbolic and religious meaning. *Lawen* cannot be separated from their sociocultural and spiritual context, which is what ultimately gives them their therapeutic value (Torri 2010). This means that while ecological studies can document the loss of biodiversity and ecosystems, they form only a part of the story. This loss causes a grief shared by other Machis from the territory (e.g., Susana et al. 2011).

## 4 Conclusions

Over the past year since our initial meeting, we have taken concrete steps together, including participating in the 2024 Turtle Island Indigenous Science conference in Canada and other scientific conferences in the United States, Chile, and Brazil to share a conversation between co‐authors of this paper about social‐ecological change in the territory and the intergenerational and intercultural challenges of equity and justice in Indigenous communities [PHJ, AMDO, IUC, BES]. We encourage our communities, especially ecologists, to create meaningful and respectful partnerships together, starting with the act of listening deeply and learning together with Two‐Eyed Seeing. From our shared work and dialog together as authors, we believe that ecologists must respond to the calls for action by supporting Indigenous Peoples in ways that align with priorities identified by their own communities.

At the same time, it must be recognized that these actions are insufficient; to truly center Indigenous TEK in ecology, systemic change is necessary to heal the harm inflicted by settler‐colonial institutions (Gazing Wolf et al. 2024). Some have argued that the slow pace of achieving trust and building respectful relationships does not align with the global capacity to avoid climate disruptions (Whyte 2020). These overwhelming realities give a sense of the immensity of the tasks that confront our communities. In the face of planetary crises, there is a real tension and contrast between the need for urgent action and the careful, trust‐building processes that are essential for genuine partnerships. How, then, do we begin? In our experiences together, we argue that precisely by deep listening and embracing the diversity of ways of knowing, we can create small, but vital, spaces of trust to create local actions and collaborations. Indeed, knowledge partnerships between Indigenous Peoples and ecologists lead to deeper and more equitable understandings of nature (Dawson et al. 2024; Molnár et al. 2024). The following three reflections emerge from our gathering in Conguillío to our extended conversations and collaboration together. They are shared as a call to reimagine the ecological sciences as a space for reciprocal engagement with Indigenous Peoples.

### 4.1 Meaningful Engagement Takes Time and Respect

Meaningful engagement must begin with intentional, respectful, and sustained listening. Respecting the time that Indigenous Peoples offer is a foundational act of reciprocity. This also involves ecologists doing the important preparatory work to review Indigenous engagement protocols, guides, and building inter‐ and trans‐disciplinary teams. To avoid malpractices, preparation matters: ecologists must “do their homework” to prepare for engagement with Indigenous Peoples. Care and time are needed to build trust and cannot be rushed (Christopher et al. 2008; Guzman and Krell 2024; Hill et al. 2020). The time and cost commitment for engagement is considerable and there are many temporal barriers as well (Castleden et al. 2012). Learning from others' experiences and following key protocols for working with Indigenous data, such as the CARE principles for Indigenous Data Governance (Carroll et al. 2020) and other good practice frameworks (Hill et al. 2020; Whyte et al. 2016) will be helpful to find the balance between communities' needs and researchers' agendas.

### 4.2 Ecologists Must Center Indigenous Peoples and Indigenous TEK in Research

Ecologists can become advocates for the involvement of Indigenous Peoples and local communities in ecological and environmental research, ensuring the research directly serves their communities, that their knowledge is respected, and that their voices are amplified within settler institutions. Learning from Indigenous TEK and using this knowledge—together—to increase biodiversity and ecosystem management may lead toward a more sustainable use of our planetary resources. This requires moving beyond ecologists' comfort zones, seeking partners and collaborators where knowledge and experiences falter, and being transparent about ecologists' limitations and challenges (see Bozhkov et al. 2020; Castleden et al. 2010). Successful knowledge partnerships are built upon respect, responsibility, reciprocity, and relevance. From our experience, what is needed is increased capacity for each party to articulate their knowledge and practices to each other through intercultural partnerships. These are supported by a foundation of good faith that honors Indigenous sovereignty, self‐governance, advocacy, and coordination (Austin et al. 2019).

### 4.3 Institutions Should Strengthen Indigenous Partnerships, Ensuring Both Meaningful Support and Institutional Accountability

As ecologists, many hold leadership roles in funding bodies, academic institutions, and other spaces where we can, and should be, vocal about better supporting underrepresented researchers, especially Indigenous scholars. Ecologists can better advocate for Indigenous Peoples' participation in ecological research, ensuring that it serves their communities, respects Indigenous TEK, and offers opportunities for them to represent themselves. Ecologists should urge scientific journals to increase visibility for local‐scale studies, requiring clear acknowledgment of Indigenous Peoples' contributions to studies conducted in their territories, including authorship and considering Indigenous data governance to protect their knowledge and data sovereignty (see Carroll et al. 2020). Our communities should encourage interdisciplinary collaborations and question the funding structures and timelines that restrict more meaningful partnerships (see Doering et al. 2022). This also means supporting and funding Indigenous Peoples and local communities in leading their own initiatives, including policy involvement, business leadership, and youth‐led efforts.

From the local to the international scale, there is increasing recognition of Indigenous TEK as essential to the protection of nature. This includes local policy frameworks such as Chile's new Biodiversity and Protected Areas Service to international policy frameworks. However, other entities and policies continue to authorize projects that directly impact nature, territories, and the lifeways of Indigenous Peoples—contradicting agreements to honor Indigenous rights. Genuine respect for the roles of Indigenous Peoples in protecting nature and their territories requires honest and transparent engagement with communities, incorporating their knowledge and values, and co‐developing sustainable pathways forward. This depends on trust‐building and common ground.

To address the biodiversity crisis, engagements between ecologists and Indigenous Peoples cannot be trapped in the same symbolic recognition or token gestures that continue to invalidate Indigenous TEK. Therefore, we must take action, individually and collectively, to reflect our shared values and responsibility for taking care of nature. From our conversations together as co‐authors, across our diverse territories and ways of knowing, Two‐Eyed Seeing provided a way of viewing different forms of knowledge as complementary towards a common vision for the future. We learned that deep listening and building respectful relationships are not a step before the work—it is an important part of the work.

## Author Contributions

**Andrea Monica D. Ortiz:** conceptualization (lead), data curation (lead), formal analysis (lead), investigation (lead), methodology (lead), project administration (lead), writing – original draft (lead), writing – review and editing (lead). **Patricia Huinca Blanco:** conceptualization (equal), methodology (supporting), validation (equal), writing – review and editing (supporting). **Carlos Alberto Arnillas:** investigation (equal), methodology (equal), writing – original draft (equal), writing – review and editing (equal). **Anni Arponen:** writing – original draft (supporting), writing – review and editing (supporting). **Marc W. Cadotte:** writing – original draft (supporting), writing – review and editing (supporting). **Javiera Beatriz Chinga Chamorro:** conceptualization (equal), methodology (equal), writing – original draft (equal), writing – review and editing (supporting). **Mariana C. Chiuffo:** conceptualization (equal), data curation (equal), formal analysis (equal), investigation (equal), methodology (equal), writing – original draft (equal), writing – review and editing (equal). **Sharon Collinge:** writing – original draft (supporting), writing – review and editing (supporting). **Kadambari Devarajan:** writing – original draft (supporting), writing – review and editing (supporting). **Ken Ehrlich:** conceptualization (equal), writing – original draft (supporting), writing – review and editing (supporting). **Marilyn Grell‐Brisk:** conceptualization (equal), writing – original draft (supporting), writing – review and editing (supporting). **Claudio Guevara:** methodology (supporting), writing – review and editing (supporting). **Rebecca W. Kariuki:** conceptualization (equal), investigation (equal), methodology (equal), writing – original draft (equal), writing – review and editing (equal). **Heather M. Kharouba:** writing – original draft (supporting), writing – review and editing (supporting). **Tara G. Martin:** writing – original draft (supporting), writing – review and editing (supporting). **Ana Carolina Prado‐Valladares:** conceptualization (equal), investigation (equal), methodology (equal), writing – original draft (equal), writing – review and editing (equal). **Helen M. Regan:** conceptualization (equal), methodology (equal), writing – original draft (equal), writing – review and editing (equal). **Nicolás Santos Domínguez:** data curation (equal), formal analysis (supporting), investigation (supporting). **Bruno Eleres Soares:** conceptualization (supporting), writing – original draft (equal), writing – review and editing (equal). **Gisela C. Stotz:** methodology (equal), writing – original draft (equal), writing – review and editing (equal). **Ivette Ulloa Caniú:** investigation (supporting), methodology (supporting). **Kristiina Visakorpi:** methodology (equal), writing – original draft (equal), writing – review and editing (equal). **Marten Winter:** writing – original draft (supporting), writing – review and editing (supporting). **Florencia A. Yannelli:** conceptualization (equal), investigation (equal), methodology (equal), writing – original draft (equal), writing – review and editing (equal).

## Conflicts of Interest

The authors declare no conflicts of interest.

## Supporting information


**Data S1:** ece371914‐sup‐0001‐Supinfo.zip.

## Data Availability

The authors have nothing to report.

## Endnotes

^1^
“Each contracting Party shall […] respect, preserve and maintain knowledge, innovations and practices of indigenous and local communities embodying traditional lifestyles relevant for the conservation and sustainable use of biological diversity […].” (Article 8(j), UN Convention on Biological Diversity). Decision 16/4 at the 16th Conference of the Parties of the United Nations Convention on Biological Diversity, Cali, Colombia (2024).

^2^

*Wallmapu* is the name of the territory of the Mapuche Indigenous Peoples. *Wall‐* means surrounding and ‐*mapu* means land or territory, in the Mapudungun language.

^3^
Mapuche traditional healer and spiritual leader. Machis are figures of spiritual authority within the Mapuche community and hold great knowledge about Mapuche medicine and spiritual activities. Many machis are women, but this role can also be held by men.

^4^
The Mapuche language.

^5^
Mapuche is an umbrella term for a diverse group of Indigenous Peoples in South‐Central Chile and parts of Argentina, including the Pehuenche (people of the pewen tree, *Araucaria araucana*), Lafkenche (people of the coast), Huilliche (people of the south), among other groups.

^6^

*Newen* is generally translated as force; it is understood as an ethereal, volitional, and highly mobile entity. *Ngen* can be translated as “spirit master,” but is related to guardianship rather than ownership. *Ngen* are thought of as entities corresponding to specific features of the landscape such as waterways or hills (di Giminiani, 2022).
