# Correction to “Listening Deeply to Indigenous People: A Collaborative Perspective and Reflection Between a Mapuche Machi and Ecologists”

**DOI:** 10.1002/ece3.72062

**Published:** 2025-08-28

Ortiz, A. M. D., P. Huinca Blanco, C. A. Arnillas, et al. 2025. “Listening Deeply to Indigenous People: A Collaborative Perspective and Reflection Between a Mapuche Machi and Ecologists.” *Ecology and Evolution* 15, no. 8: e71914. https://doi.org/10.1002/ece3.71914.

On Page 7, in the first paragraph, a section of the Acknowledgments has jumped to the text. This obfuscates the meaning of the sentence.

“She wonders whether this is truly due to “climate change”, Although she was unable to be listed as a co‐author, she shares a personal commitment to the collaboration. or rather, the impacts of extractive industries in the territory that have used all the water and threaten to harm the river more with the hydroelectric plant.”

Should read as “She wonders whether this is truly due to ‘climate change,’ or rather, the impacts of extractive industries in the territory that have used all the water and threaten to harm the river more with the hydroelectric plant.”

We apologize for this error.

