# Supplementary figures and images for: Listening Deeply to Indigenous People: A Collaborative Perspective and Reflection Between a Mapuche Machi and Ecologists
Source: Ecol Evol. 2025 Aug 7;15(8):e71914. doi: 10.1002/ece3.71914 (PMC12329347; doi:10.1002/ece3.71914)

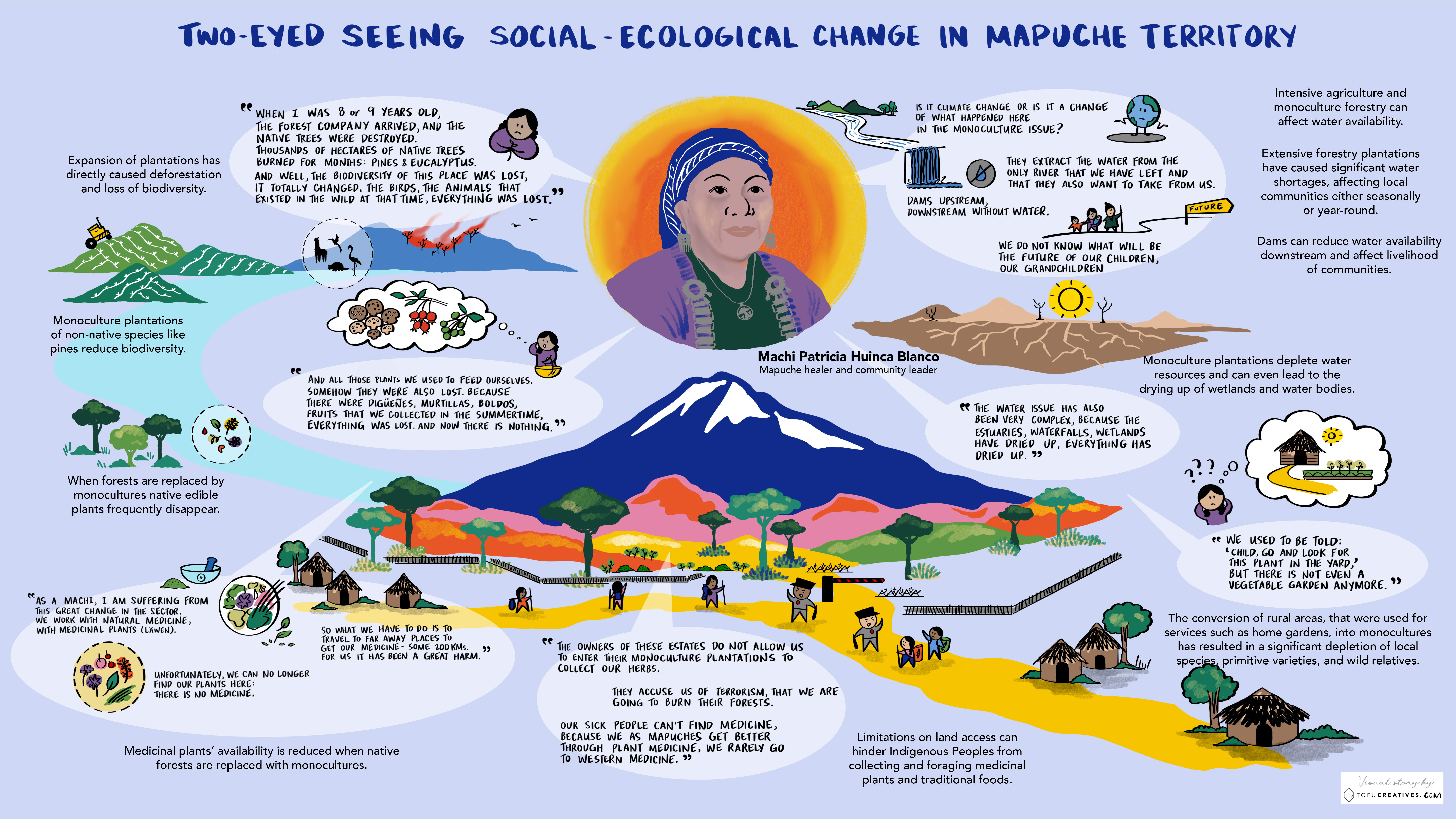

Supplement: Supplementary file 1 — Data S1: ece371914‐sup‐0001‐Supinfo.zip. [file ECE3-15-e71914-s001.zip › 2024_Mapuche_V4.jpg]

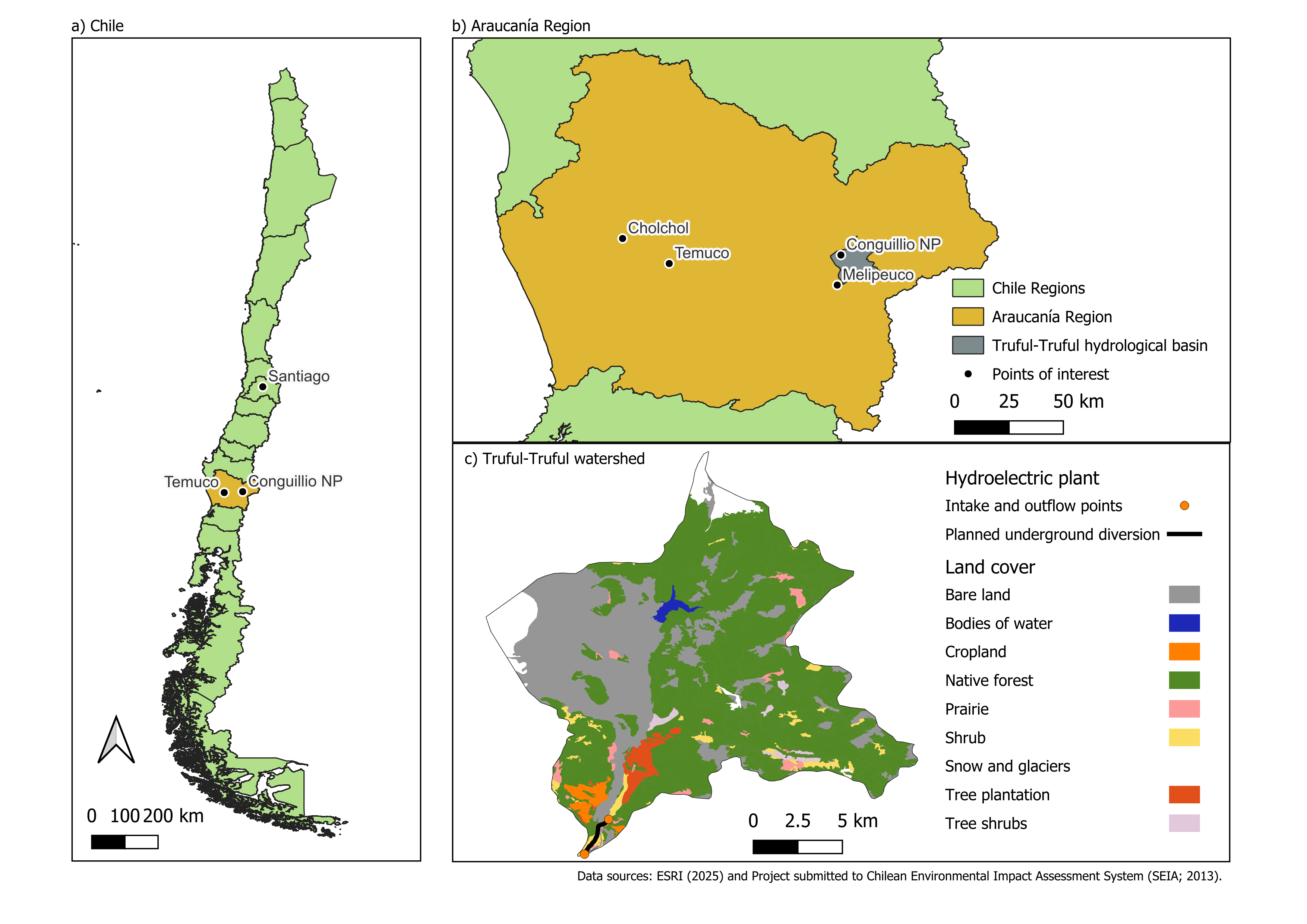

Supplement: Supplementary file 1 — Data S1: ece371914‐sup‐0001‐Supinfo.zip. [file ECE3-15-e71914-s001.zip › FIG1.png]

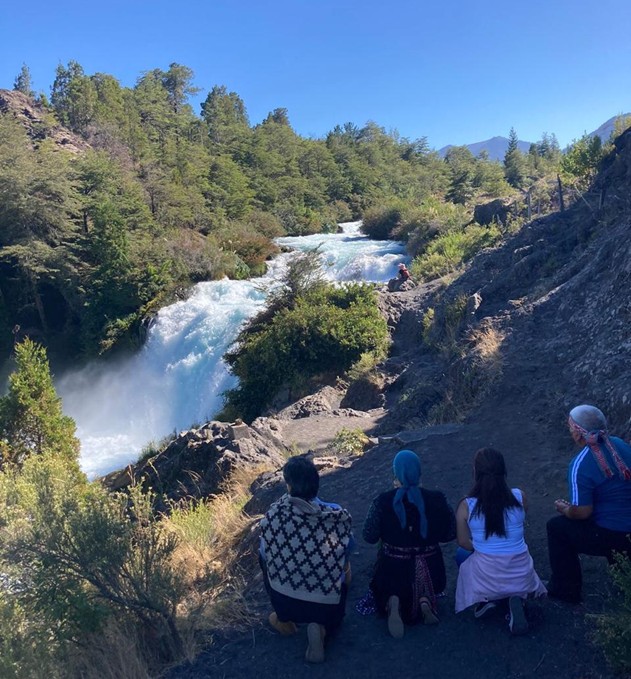

Supplement: Supplementary file 1 — Data S1: ece371914‐sup‐0001‐Supinfo.zip. [file ECE3-15-e71914-s001.zip › FIG2.jpg]
